# Pathogenomics analysis of high-risk clone ST147 multidrug-resistant *Klebsiella pneumoniae* isolated from a patient in Egypt

**DOI:** 10.1186/s12866-024-03389-z

**Published:** 2024-07-10

**Authors:** Fatma A. Elgayar, Mona K. Gouda, Alaa Aboelnour Badran, Nancy M. El Halfawy

**Affiliations:** 1 Department of Botany and Microbiology, Faculty of Science, Alexandria University, Moharram Bek 21511, Alexandria, Egypt; 2 Department of Clinical Pathology, Faculty of Medicine, Mansoura University, Mansoura, Egypt

**Keywords:** *Klebsiella pneumoniae*, Resistome, Virulence, High-risk clone ST147, Multidrug resistant (MDR), Whole-genome sequencing (WGS), Horizontal gene transfer (HGT)

## Abstract

**Background:**

The emergence of multi-drug-resistant *Klebsiella pneumoniae* (MDR-KP) represents a serious clinical health concern. Antibiotic resistance and virulence interactions play a significant role in the pathogenesis of *K. pneumoniae* infections. Therefore, tracking the clinical resistome and virulome through monitoring antibiotic resistance genes (ARG) and virulence factors in the bacterial genome using computational analysis tools is critical for predicting the next epidemic.

**Methods:**

In the current study, one hundred extended spectrum β-lactamase (ESBL)-producing clinical isolates were collected from Mansoura University Hospital, Egypt, in a six-month period from January to June 2022. One isolate was selected due to the high resistance phenotype, and the genetic features of MDR-KP recovered from hospitalized patient were investigated. Otherwise, the susceptibility to 25 antimicrobials was determined using the DL Antimicrobial Susceptibility Testing (AST) system. Whole genome sequencing (WGS) using Illumina NovaSeq 6000 was employed to provide genomic insights into *K. pneumoniae* WSF99 clinical isolate.

**Results:**

The isolate *K. pneumoniae* WSF99 was phenotypically resistant to the antibiotics under investigation via antibiotic susceptibility testing. WGS analysis revealed that WSF99 total genome length was 5.7 Mb with an estimated 5,718 protein-coding genes and a G + C content of 56.98 mol%. Additionally, the allelic profile of the WSF99 isolate was allocated to the high-risk clone ST147. Furthermore, diverse antibiotic resistance genes were determined in the genome that explain the high-level resistance phenotypes. Several β-lactamase genes, including *bla*_CTX−M−15_, *bla*_TEM−1_, *bla*_TEM−12_, *bla*_SHV−11_, *bla*_SHV−67_, and *bla*_OXA−9_, were detected in the WSF99 isolate. Moreover, a single carbapenemase gene, *bla*_NDM−5_, was predicted in the genome, positioned within a mobile cassette. In addition, other resistance genes were predicted in the genome including, *aac*(6’)-Ib, *aph*(3’)-VI, *sul1*, *sul2*, *fosA*, *aadA*, *arr-2*, *qnrS1*, *tetA* and *tetC*. Four plasmid replicons CoIRNAI, IncFIB(K), IncFIB(pQil), and IncR were predicted in the genome. The draft genome analysis revealed the occurrence of genetic mobile elements positioned around the ARGs, suggesting the ease of dissemination via horizontal gene transfer.

**Conclusions:**

This study reports a comprehensive pathogenomic analysis of MDR-KP isolated from a hospitalized patient. These findings could be relevant for future studies investigating the diversity of antimicrobial resistance and virulence in Egypt.

## Background

Antimicrobial resistance (AMR) is a major clinical health challenge that is anticipated to cause 10 million deaths by 2050, greatly exceeding deaths from cancer patients [1, 2]. AMR is the consequence of a number of factors, including poverty, inappropriate antibiotic prescriptions, uncontrolled antimicrobial agent consumption, and a lack of effective nosocomial infection prevention measures [3]. There is an opportunity that surviving pathogens could acquire resistance when exposed to subtherapeutic antibiotic concentrations [4]. Therefore, healthcare has a significant role in raising awareness and preventing the emergence and dissemination of AMR.

*Klebsiella pneumoniae* is one of the most prevalent multidrug-resistant (MDR) pathogens that harbors antibiotic-resistant encoding genes (ARGs) [5]. Several nosocomial infections, including pneumonia, urinary tract infections, septicemia, and meningitis, are thought to be caused by *K. pneumoniae* [6, 7]. It is also associated with a high mortality rate due to the scarcity of efficient therapeutic options [8, 9]. Thus, the World Health Organization (WHO) has designated *K. pneumoniae* as a species of high priority due to the growing concern about AMR [10]. Therefore, *K. pneumoniae* has several mechanisms for AMR, that play a crucial role in the emergence of MDR strains [11, 12]. For instance, *K. pneumoniae* possesses miscellaneous antibiotic resistance genes, including extended spectrum β-lactamase (ESBL) genes and carbapenemase-encoding genes [13, 14]. Furthermore, *K. pneumoniae* exhibits a number of virulence factors essential for pathogenicity, including capsule production, lipopolysaccharide, and iron acquisition systems [15].

Recently, Egypt witnessed a widespread prevalence of MDR-KP infections due to the repetitive use of β-lactam and carbapenemase antibiotics in therapeutic regimens for infection [16, 17]. However, controlling these life-threating infections is becoming more challenging considering the rapid dissemination of resistance genes among MDR-KP and the lack of information regarding the genomic features [17]. Thus, periodic surveillance of healthcare settings in Egypt is essential to fill the gap by investigating the antimicrobial resistance mechanisms, virulence determinants, and epidemiology of *K. pneumoniae* [17, 18]. Indeed, whole genome sequencing (WGS) is a potent tool in the fight against infections in healthcare environments and discriminates between lineages that cause infection [19]. It contributes significantly to the low-cost production of millions of reads in a single run [20]. Consequently, this allows for tracing and identifying unanticipated modes of antibiotic resistance mechanisms and transmission in the MDR strains that revealed resistance to a broad range of antimicrobial agents [21]. The data obtained from WGS provides a better understanding of the bacterial dissemination of them in Egypt and offers treatment options.

The current work uses WGS-based analyses to gain insights into the resistome, virulome, and MGEs in the draft genome of *K. pneumoniae* WSF99, isolated from a wound aspiration of a 64-year-old diabetic cardiac female patient suffering from uterine adenoma in Egypt. Moreover, this study discerns the relatedness of the MDR-KP WSF99 isolate to other reported genomes.

## Materials and methods

### Sample collection and bacterial isolation

Isolate was obtained from the Clinical Pathology Department, Faculty of Medicine, El-Mansoura University (Egypt) in six months, starting from January 2022 to June 2022. This study was performed based on hospital ethical guidelines as previously approved by the University Institutional Review Board of Mansoura University, Egypt (approval number R.23.12.2423). Isolation was conducted from the wound aspirations of a female patient using MacConkey agar plates (MAC; SRL, India), then incubated for 24–48 h at 37 °C. Preliminary identification using Gram staining, coagulase, catalase, oxidase, and the IMViC test was accomplished. Moreover, single colonies were streaked on MacConkey agar (MAC; SRL, India) for purification. Pure cultures were stored at -20 °C in tryptic soy broth (TSB; SRL, India) supplemented with 50% (v/v) glycerol for further investigation.

### Identification and antimicrobial susceptibility testing (AST)

Further identification at species-level using the VITEK 2 compact system (BioMérieux, France; http://www.biomerieux.com) was performed according to the manufacturer’s instructions. Moreover, antimicrobial susceptibility testing of the isolate was performed using the DL Antimicrobial Susceptibility Testing (AST) system (Zhuhai DL Biotech, China; https://en.medicaldl.com). Bacterial suspension equivalent to 0.5 McFarland turbidity was prepared, and 50 µL of suspension was subjected to Enterobacteriaceae AST CARD (DL-120E) per well. The results were obtained after 24 h against 25 antimicrobial agents, namely, cefazolin, gentamicin, ampicillin, imipenem, ertapenem, piperacillin-tazobactam, trimethoprim-sulfamethoxazole, cefepime, cefuroxime, cefotaxime, cefoxitin, levofloxacin, ampicillin-sulbactam, meropenem, amikacin, ceftazidime, chloramphenicol, nitrofurantoin, polymyxin B, minocycline, ceftazidime-clavulanate, cefotaxime-clavulanate, cefoperazone-sulbactam, tigecycline, and azithromycin. The minimum inhibitory concentrations (MIC, µg/mL) of the isolate were interpreted as susceptible (S), intermediate (I), or resistant (R) according to Clinical and Laboratory Standards Institute (CLSI) guidelines [22].

### Whole genome sequencing (WGS)

Genomic DNA was extracted from WSF99 isolate grown overnight in TSB using the GeneJET Genomic DNA Purification Kit (Thermo Fisher Scientific, UK) following the manufacturer’s instructions and eluted in 10 mM Tris-HCl (pH 8.0). Libraries were prepared using the Nextera XT Library Prep Kit (Illumina, USA) on a Hamilton Microlab STAR automated liquid handling system (Hamilton Bonaduz AG, Switzerland) following the manufacturer’s protocol. Whole genome sequencing (WGS) was outsourced and undertaken by MicrobesNG in July 2023 (Birmingham, UK; http://microbesng.uk) on an Illumina NovaSeq 6000 platform (Illumina, USA) using a 250 bp paired end protocol with 30X sequence coverage.

### Genome assembly and annotation

Reads were trimmed using Trimmomatic (Version 0.30) [23] with a sliding window quality cutoff of Q15. *De novo* assembly of reads was performed using SPAdes (Version 3.7) [24]. Contigs were annotated using Prokka software (Version 1.11) [25] and the NCBI Prokaryotic Genome Annotation Pipeline (PGAP) [26]. BV-BRC web server (Version 3.30.19*a*; https://www.bv-brc.org/) identified protein-encoding regions and assigned functions to the genes, rRNA, tRNA, and subsystems in the genome. Metabolic pathways were predicted using Kyoto Encyclopedia of Genes and Genomes (KEGG; http://www.genome.jp/kegg) [27] (accessed on October 2023). Clusters of Orthologous Groups (COG) functional categories were predicted using egg-NOG Mapper (http://eggnog-mapper.embl.de) [28]. CGView sever (https://proksee.ca) generated the circular genome’s graphical map [29].

### Phylogenomic and pan-genome analyses

Whole genome-based phylogeny analysis was performed using the Type (Strain) Genome Server (TYGS; https://tygs.dsmz.de) [30] with closely related *K. pneumoniae* strains (Table 1) obtained from BV-BRC database (accessed on September 2023). The phylogenomic tree was reconstructed using FastME 2.1.6.1 [31] from Genome BLAST Distance Phylogeny (GBDP) distances calculated from genome sequences under the algorithm “coverage” and distance formula *d5.* The tree was rooted at the midpoint and visualized with PhyD3 [32]. Pan genome analysis was performed using the Integrated Prokaryotes Genome and Pan-Genome Analysis service (IPGA v1.09; https://nmdc.cn/ipga/) [33].

**Table 1:** *Klebsiella pneumoniae* strains used in this study including GenBank accession number

Strain	Accession number	MLST	Geographical location	Year
WFS99	JAWIZL000000000	ST147	Egypt	2022
YNK-2023	JASMSL000000000	ST147	Egypt	2020
938	PYWE00000000	ST147	India	2015
Rize-53-TR	JABMCK000000000	ST147	Turkey	2015
C5-E1-13	MSYW00000000	ST147	Tunisia	2016
ATH9	VJXN00000000	ST147	Greece	2016
VB4048	JAHXZP000000000	ST147	India	2018
KLB_MDR_390314	CP133011	-	India	2023
Bio73	CP093852	ST 2096	Turkey	2016
KE3783	VNMC00000000	ST152	Germany	2022
P30-63	JAHVFW000000000	ST307	Malawi	2016
KP1221	JAVBIU000000000	ST307	Pakistan	2020
YMC2016/02/N207	SSKH00000000	ST307	South Korea	2016
1-G9	JAHUYY000000000	ST307	Spain	2020
BL37-2	VIDD00000000	ST37	China	2016
EN5289	JAELUW000000000	ST11	India	2016
Ecl_5.VN	JAJCTX000000000	ST340	South Africa	2021
P30-67	JAHVFV000000000	ST340	Malawi	2016
CRKP-20	JAJSBW000000000	ST11	China	2020
WYKP587	JASATY000000000	ST11	China	2022
XHKPN396	JAEOAM000000000	ST11	China	2018

### Identification of MLST, virulence, capsule, heavy metal, and antimicrobial resistance genes

Multi-locus sequence type (MLST) and heavy metal resistance genes for the isolate were determined using the Institute Pasteur website (https://bigsdb.pasteur.fr/klebsiella/) (accessed on November 2023). Virulence factors in the genome were investigated using the Institute Pasteur website (https://bigsdb.pasteur.fr/klebsiella/) and VFanalyzer platform (https://www.mgc.ac.cn), available through the Virulence Factor Database (VFDB; accessed on May 2024). Kaptive Web was used for capsular (K) and lipopolysaccharide (O-antigen) locus typing (https://kaptive-web.erc.monash.edu), accessed on August 2023 [34]. Heavy metal resistance genes were predicted using the Institute Pasteur website (https://bigsdb.pasteur.fr/klebsiella/). Antibiotic resistance genes (ARG) were predicted using the Comprehensive Antibiotic Resistance Database (CARD; https://card.mcmaster.ca/) [35] and ResFinder (Version 4.3.3) available through Center for Genomic Epidemiology (CGE; https://cge.cbs.dtu.dk) (accessed on August 2023).

### Detection of genetic mobile elements (IS, plasmids, ICE, prophages)

Insertion sequences (IS) and transposons were predicted with ISfinder server (https://www-is.biotoul.fr) [36] using BLASTn (Version 2.2.31+). Plasmids were detected by the PlasmidFinder (Version 2.0) online tool [37] provided by the CGE (accessed on August 2023). Integrative and conjugative elements (ICEs) were identified by the ICEfinder web-based tool from the ICEberg (Version 2.0) [38], with an e-value of 1e-150 (accessed on September 2023). Prophage sequences were identified using the PHAge Search Tool Enhanced Release web server (PHASTER; https://phaster.ca) [39].

## Results

### Biochemical characterization and AST

Strain WSF99 was isolated from the wound aspiration of a 64-year-old diabetic cardiac female patient suffering from uterine adenoma who stayed at the hospital for 18 days and died due to cardiac arrest. The strain was identified biochemically by 99% probability as *Klebsiella pneumoniae* using the VITEK 2 system. The MICs were interpreted (Table 2) according to CLSI guidelines, and strain WSF99 was found to be resistant to all antibiotics under investigation. For more in-depth resistome and virulome analyses, the strain, namely *K. pneumoniae* WSF99, was subjected to next-generation sequencing.

**Table 2:** Minimal inhibitory concentrations (MICs) interpretation of *Klebsiella pneumoniae* WSF99 as per CLSI 2021 guidelines

Antibiotic class	Antibiotics	Susceptibility (Interpretation*)	MIC values (µg/mL)
Aminoglycosides	Gentamicin	R	>=16
	Amikacin	R	>=64
β-Lactam antibiotics	Ampicillin-sulbactam	R	>=32/16
	Ceftazidime-clavulanate	R	>=2/8
	Cefotaxime-clavulanate	R	>=2/8
	Cefoperazone-sulbactam	R	>=128/64
	Piperacillin-tazobactam	R	>=128/4
Carbapenems	Ertapenem	R	>=16
	Imipenem	R	>=32
	Meropenem	R	>=32
Cephalosporins	Cefazolin	R	>=32
	Cefuroxime	R	>=64
	Cefotaxime	R	>=64
	Ceftazidime	R	>=32
	Cefepime	R	>=32
Penicillin	Ampicillin	R	>=32
	Cefoxitin	R	>=64
Fluoroquinolones	Levofloxacin	R	>=8
Macrolides	Azithromycin	R	>=64
Chloramphenicol	Chloramphenicol	R	= 16
Polymyxins	Polymyxin B	R	<=1
Tetracyclines	Minocycline	R	>=16
	Tigecycline	R	= 2
Sulphonamide	Trimethoprim-sulfamethoxazole	R	>=8/152

*R, resistant

### Genome assembly features

**Fig. 1:**
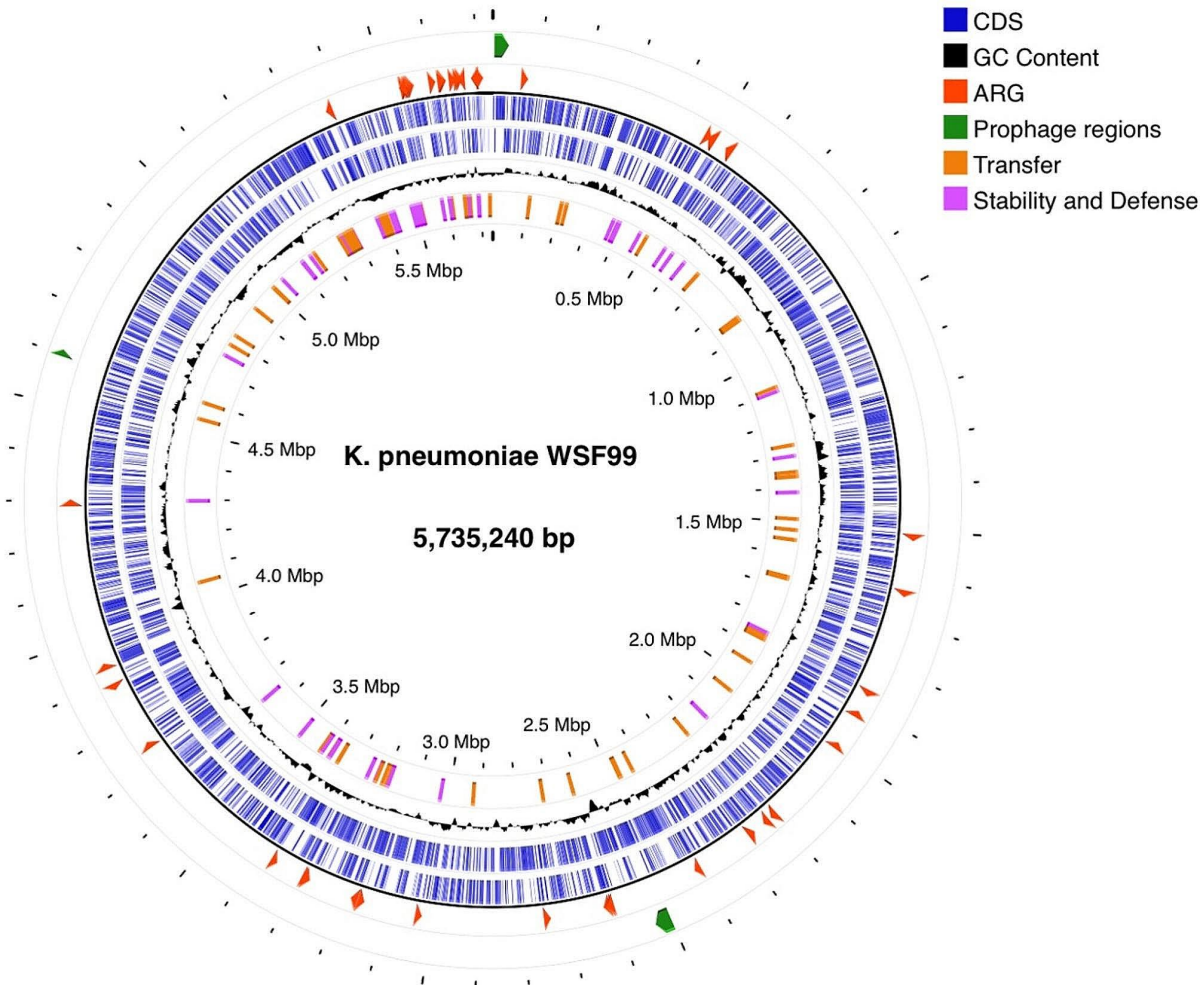
Circular draft genomic map of the *Klebsiella pneumoniae* WSF99 contig sequences. The genome is 5,735,240 bp in size and has a G + C content of 56.98%. The ARG genes are denoted as red arrows, prophage regions are denoted as green arrows, transfer genes are denoted in orange arrows, and genes related to defense and stability are denoted in pink arrows. Image was generated using the Proksee web server (https://proksee.ca/)

General genomic features of *K. pneumoniae* strain WSF99 draft genome (NCBI accession number JAWIZL000000000) were obtained using the Illumina NovaSeq platform, yielding 937,218 reads with a median insert size of 721 bases and contigs with an N50 value of 329,042 bp. The total genome length was 5.7 Mb, with an estimated 5,718 CDS regions and a G + C content of 56.98 mol% (Fig. 1). A total of 86 tRNA genes, and 17 rRNA genes were predicted using the BV-BRC server. MLST allelic analysis was performed *in silico* using seven housekeeping genes (*gapA*, *infB*, *mdh*, *pgi*, *phoE*, *rpoB*, *tonB*). Therefore, isolate WSF99 was assigned to sequence type ST147, and the allelic profile was 3-4-6-1-7-4-38.

### COG functional categories prediction and subsystem analysis

**Fig. 2:**
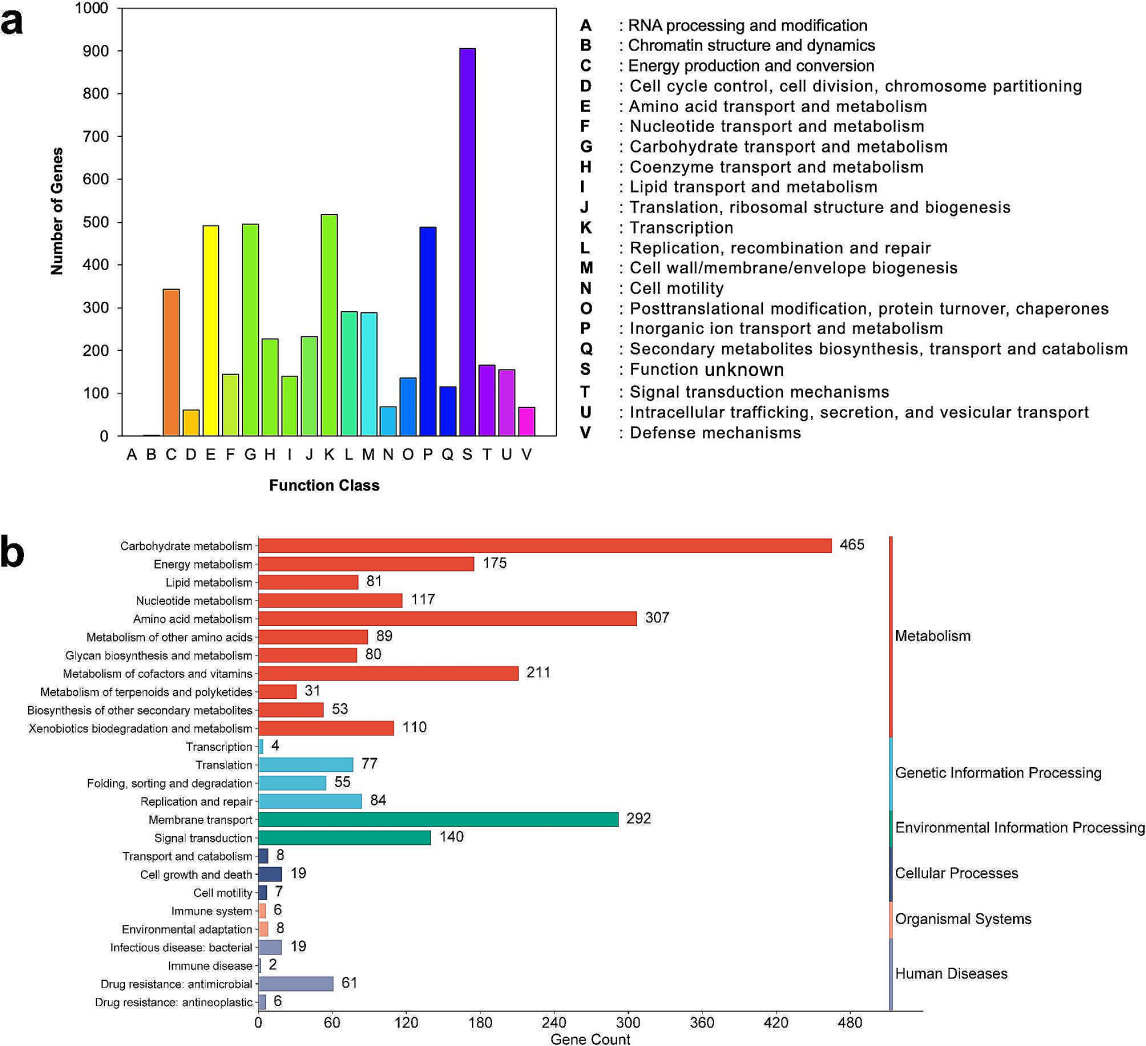
(**a**) Cluster of orthologous groups (COG) classification of protein functions (**b**) KEGG pathway classification map of *Klebsiella pneumoniae* WSF99. Genes were classified into six categories (Metabolism, Genetic Information Processing, Environmental Information processing, Cellular Processes, Organismal systems and Human Diseases) according to the biological pathways

The clusters of COGs in the WSF99 genome were predicted with eggNOG-mapper and assigned to 21 functional categories (Fig. 2a). Furthermore, the genome annotations obtained using the KEGG server were used to generate an overview of the subsystem categories and feature distribution of the genome under investigation. The distribution of GO revealed that metabolism categories were the most abundant. Furthermore, genes related to membrane transport were among the most abundant in the environmental information processing category. Otherwise, KEGG assigned the WSF99 isolate to human disease pathways. An overview of the genome’s subsystem annotations is provided in Fig. 2b.

### Phylogenomic and pan-genome analyses

**Fig. 3:**
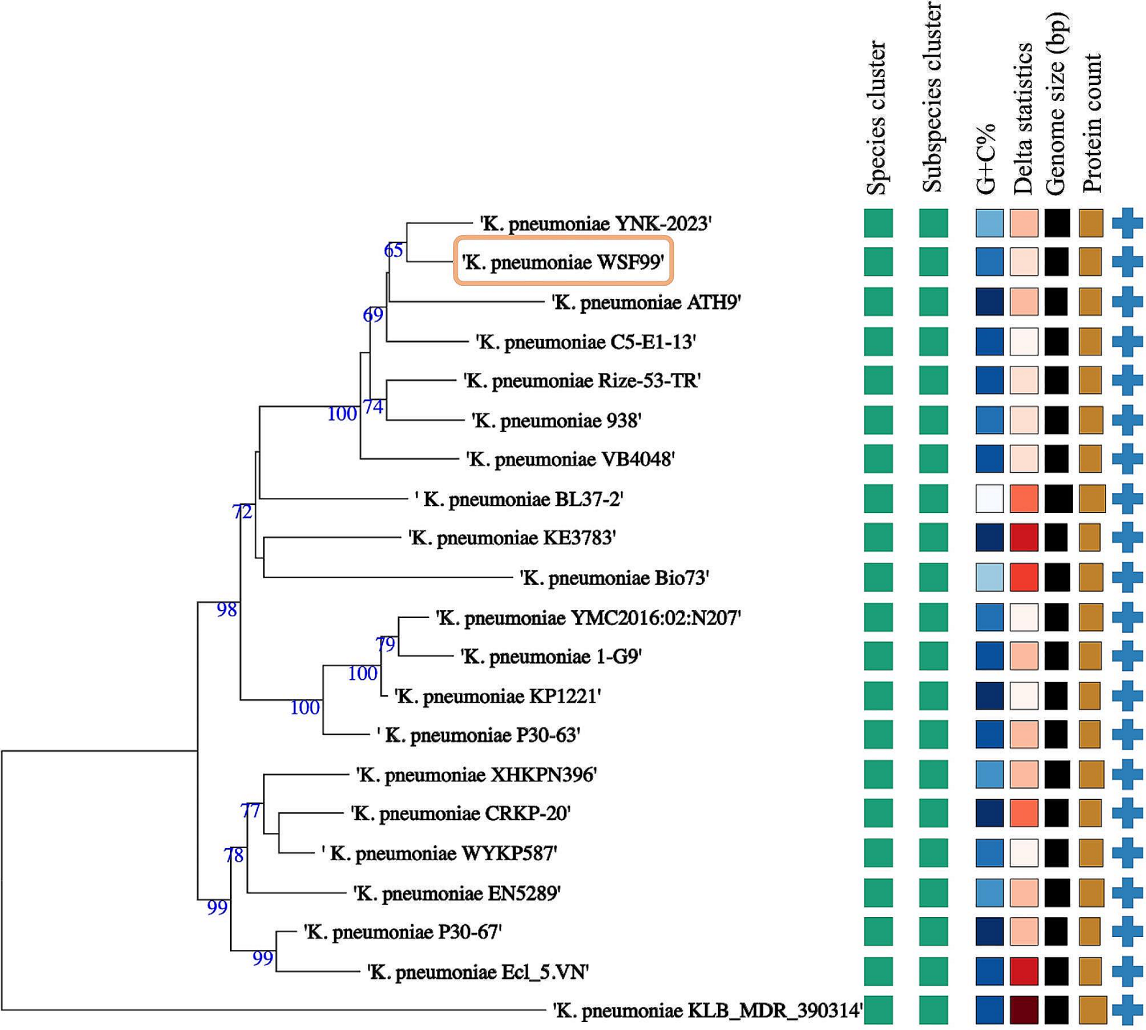
Phylogenomic analysis of *Klebsiella pneumoniae* WSF99. Tree inferred with FastME 2.1.6.1 from GBDP distance calculated from genome sequences. Branch lengths were scaled in terms of the GBDP distance formula *d5*. The numbers above the branches are GBDP pseudo-bootstrap support values > 60% from 100 replications, with an average branch support of > 60%. The tree was rooted at the midpoint. Leaf labels with different colours indicate species and subspecies clusters. The tree was constructed with the TYGS webserver (https://tygs.dsmz.de/)

**Fig. 4:**
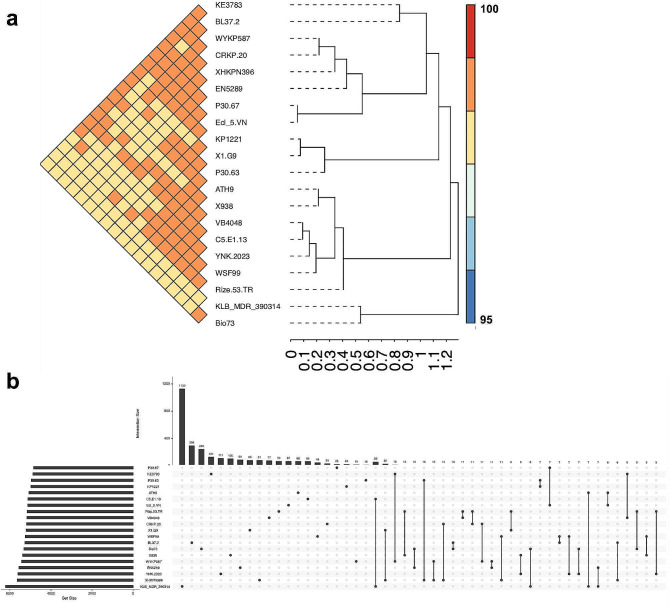
(**a**) Pairwise comparison of average nucleotide identity (ANI) (**b**) Upset figure showing the unique genes of each strain

A whole genome-based taxonomic analysis was performed to obtain insights into the phylogenetic relationship between *K. pneumoniae* strain WSF99 and other twenty *K. pneumoniae* strains (Fig. 3). The results revealed that *K. pneumoniae* strain WSF99 is closely related to MDR *K. pneumoniae* strain YNK-2023 (accession number JASMSL000000000), previously isolated from a hospital respiratory patient in Egypt. Moreover, WSF99 isolate was clustered together with strains with ST147, including strains ATH9 (VJXN00000000; Greece), C5-E1-13 (MSYW00000000; Tunisia), Rize-53-TR (JABMCK000000000; Turkey), 938 (PYWE00000000; India), and VB4048 (JAHXZP000000000; India). The average nucleotide identity (ANI) values of WSF99 and 20 other *K. pneumoniae* strains were calculated (Fig. 4a), and the ANI value with *K. pneumoniae* strain YNK-2023 was the highest (99.81%). Pan-genome analysis revealed that the unique genes in each genome ranged from 16 to 1130 (Fig. 4b), and the genome of WSF99 exhibited 48 unique genes.

### Antibiotic and heavy metal resistance genes analyses

Isolate WSF99 displayed a high degree of antimicrobial resistance to multiple β-lactam antibiotics, including *bla*_CTX−M−15_, *bla*_TEM−1_, *bla*_TEM−12_, *bla*_SHV−11_, *bla*_SHV−67_, and *bla*_OXA−9_(Table 3). Moreover, one carbapenemase gene, *bla*_NDM−5_, was predicted in the genome and partitioned into a mobile cassette arranged as IS*30*-IS*630*-*bla*_NDM−5_-IS*26*-Tn*As3*-IS*5075*. Meanwhile, the *bla*_CTX−M−15_ gene was bracketed by transposon Tn3 and the insertion sequence IS*Ecp1*. In addition, other resistance genes were predicted in the genome, including aminoglycosides (*aadA1, aac*(6’)-Ib, *aph(3’)-*Ia and *aph*(3’)-VI), fluoroquinolones (*oqxA*, *oqxB*, *qnrS*1), trimethoprim (*dfrA1*, *dfrA14*), fosfomycin (*fosA*), sulfonamides (*sul1*, *sul2*), and tetracyclines (*tetA*, *tetC*). Otherwise, the isolate harbored numerous MDR efflux pumps that encoded the resistance of several antibiotics’ families. For instance, Resistance Nodulation Division (RND) efflux system (*acrA*, *acrB, oqxA*, *oqxB*) and the multidrug efflux system (*mdtA*, *mdtB*, *mdtC*). Furthermore, heavy metal resistance genes coding for silver (*silA*, *silR*) and tellurium (*terA, terB, terC, terD, terE, terW, terX*, *terY*) resistance were predicted in the WSF99 genome.

**Table 3:** Genomic features of *Klebsiella pneumoniae* WSF99 strain clone ST147 isolated from patient in Egypt

ST	Capsular type	Antimicrobial Resistance		Virulence		Plasmid Replicon	Prophage	ICE
		Antimicrobial Class	Antimicrobial Resistance Genes	Virulence factors	Virulence Genes			
147	KL35/O5	β-lactamase	*bla*_CTX−M−15_ *bla*_TEM−1_ *bla*_TEM−12_ *bla*_SHV−11_ *bla*_SHV−67_ *bla*_OXA−9_	Regulator of mucoid phenotype A Type III fimbriae	*rmpA, rmpA2* *mrkABCDFHIJ*	ColRNAI IncFIB(K)	Klebsi-ST147-VIM1phi7.1	T4SS *virB*
		Carbapenemase	*bla*_NDM−5_	Type I fimbriae	*fimABCDEFGHIK*		Pseudo-phiPSA1	Putative IME
		Aminoglycoside	*aadA1* *aac*(6’)-Ib *arr-2* *aph*(3’)*-*Ia *aph*(3’)-VI	Aerobactin	*iucABCD* *iutA*	IncFIB(pQil)		
		Sulfonamides	*sul1*, *sul2*	Siderophore	*entABCDEFS* *fepABCDG*	IncR		
		Fluoroquinolones	*oqxA*, *oqxB* *qnrS1*	Salmochelin	*iroEN*			
		Tetracyclines	*tetA*, *tetC*	Secretion system T6SS-I	*tssCDFGHJKLM* *ompA*			
		Macrolide	*mphA*	Secretion system T6SS-II	*clpV*			
		Rifamycin	*arr-2*	Secretion system T6SS-III	*dotU*, *icmF*, *vgrG*, *sciN* *impAFGHJ*			

### Virulence and capsular genes analysis

Screening the genome revealed the occurrence of virulence genes associated with pathogenicity, which supports the concept that the WSF99 strain is a highly virulent strain. Regulator of mucoid phenotype A genes (*rmpA* and *rmpA2*) were predicted in the WSF99 genome, which confers a hypervirulent phenotype. Furthermore, the *fim* genes cluster *(fimA, fimB, fimC, fimD, fimE, fimF, fimG, fimH, fimI*, *fimK*) responsible for the production of type 1 fimbriae associated with adherence was predicted in the genome. Otherwise, *mrk* genes *(mrkA, mrkB, mrkC, mrkD, mrkF, mrkH, mrkI, mrkJ)* encoding type 3 fimbriae were identified. Moreover, fimbrial adherence determinants (*steB*) and type IV pili (*pilW*) genes were found in the genome. Other virulence factors encoding enterobactin synthesis (*entA*, *entB*, *entC*, *entD*, *entE*, *entF*, *entS*) and (*fepA, fepB*, *fepC*, *fepD*, *fepG*) which acquire iron from host cells, were determined. Additionally, the genome carried genes associated with aerobactin synthesis (*iucA, iucB, iucC*, *iucD*). Multiple secretion systems were predicted in the genome, including T6SS-I and T6SS-III. Moreover, genes *rcsAB* and *iroEN* were also predicted in the genome. In addition, capsular typing via *wzi* and *wzc* allele sequences revealed that *K. pneumoniae* WSF99 is K-type (K35), O-antigen type (O5), and the wzi23 allele was identified in the genome.

### Detection of genetic mobile elements (IS, plasmids, prophages, ICE)

Insertion sequence IS*26* belonging to the IS*6* family, associated with the mobilization of several antibiotic resistance genes in clinical isolates, was the most frequent in the WSF99 genome. Moreover, IS*Kpn25*, IS*Kpn26*, IS*903B*, and IS*Kpn14* were also predicted in the genome to be involved in colistin resistance. Additionally, other insertion sequences, namely, IS*5075*, IS*Kpn19*, IS*6100*, IS*Kpn43*, IS*102*, ISEc9, IS*Kpn24*, ISSen4 and IS*Kpn28*, were predicted in the genome. Otherwise, four plasmid replicons were predicted in the genome, namely CoIRNAI (accession number DQ298019), IncFIB(K) (accession number CP011596), IncFIB(pQil) (accession number JN233705), and IncR (accession number DQ449578). IncR harbored genes that encoded *tetA*, *dfrA1*, *aph(3’)-Ia*, *sul2*, and *qacE*. Otherwise, IncFIB(K) carried genes that encoded for insertion sequences such as IS*903* and IS*26*. Furthermore, the plasmid IncFIB(pQil) sequence contained *bla*_TEM-1_ gene and the insertion sequence IS*Kpn25*. Moreover, two bacteriophages were determined, one intact phage (PHAGE_Klebsi_ST147_VIM1phi7.1; 33.3 kb) and one questionable phage (PHAGE_Pseudo_phiPSA1; 26.8 kb). Additionally, two putative ICE elements of 84,464 and 78,538 bp length were detected in the WSF99 genome sequence.

## Discussion

*K. pneumoniae* is a problematic bacterial pathogen that exhibits MDR pattern and is associated with a high rate of mortality in hospitalized patients in the clinical world [40]. WGS approach is necessary to provide high-resolution genomic information to predict drug resistance and to decipher the dynamics of risk factor transmission [41, 42]. Thus, next-generation sequencing-facilitated resistance gene detection has the potential to prompt treatment making-decisions and eradicate pathogen infection [43]. In this study, we provide genomic insights into the emergence of the MDR-KP strain isolated from a diabetic cardiac female patient and emphasizes the transmission potential of this highly resistant pathogen. Hopefully, this information is important in reducing the dissemination of MDR-KP in Egypt.

WGS revealed that the genome size was similar to the reference genomes available in the NCBI database [17, 44]. Moreover, COG and KEGG functional annotations of the *K. pneumoniae* WSF99 revealed the genomic plasticity of this strain, which contributes significantly to its survival and pathogenicity. In this study, genes encoding carbohydrate metabolism were the most abundant in the genome, which is in agreement with a previous report [45]. Otherwise, combining antibiotic sensitivity testing with genome based ARGs analysis of WSF99 provided high confidence regarding antibiotic susceptibility. Notably, when investigating the MDR profile of *K. pneumoniae* WSF99, high levels of AMR were observed phenotypically. Moreover, the phenotypic resistance of WSF99 strain to β-lactams and carbapenems was validated by the occurrence of *bla*_CTX−M−15_, *bla*_TEM−1_, *bla*_TEM−12_, *bla*_SHV−11_, *bla*_SHV−67_, *bla*_OXA−9_, and *bla*_NDM−5_ genes in the genome sequence, respectively.

Furthermore, the phylogenomic analysis of WSF99 strain revealed that it occurs within a clade of other *K. pneumoniae* strains, clone ST147 and closely related *K. pneumoniae* strain YNK-2023, previously isolated from a hospital respiratory patient from Egypt in 2020 (accession number JASMSL000000000). ST147 is a high-risk *K. pneumoniae* clone that poses a significant risk to public health and has caused numerous outbreaks in Italy, India, Greece, and Northern African countries [46, 47]. Otherwise, according to a recent global surveillance program, the Middle East and Africa had the highest prevalence of ESBL non-carbapenem-resistant Enterobacteriaceae from 2015 to 2019 [48]. Similar to a previous report, the highest number of ARGs were detected in strains belonging to ST147 especially ESBL types (SHV, CTX-M, and TEM), which is in agreement with our results [49]. It was found that *bla*_CTX−M−15_ was the most common type and renders high resistance to different β-lactam agents and has been widely distributed in clinical isolates since 2013 [49, 50]. In accordance with a previous study, the occurrence of *bla*_CTX−M−15_, in the chromosome is responsible for forming stable MDR phenotypes [51]. Meanwhile, in the WSF99 genome, the *bla*_CTX−M−15_ gene was found downstream of the transposase IS*Ecp1*, which provides the promotor required for the expression of this ESBL gene [52]. A previous study reported that 82.35% of *K. pneumoniae* isolated from intensive care unit (ICU) patients in Alexandria, Egypt (2020) was extensively drug resistant, and about 94.12% of the isolates were ESBL-producers with *bla*_CTX−M−15_ (64.71%) [17]. Further study reported the occurrence of multiple ESBL-ARGs, including *bla*_TEM−1_, *bla*_OXA−9_ and *bla*_CTX−M−15_ in *K. pneumoniae* isolated from patients in Assiut, Egypt (2021) [15]. Thus, it could be potentially concluded that ESBL-producing *K. pneumoniae* have become more prevalent in hospital settings in Egypt.

The New Delhi metallo-β-lactamase gene (*bla*_NDM_), which confers enhanced hydrolytic activity against carbapenems, was the predominant gene correlated with carbapenem resistance. It is globally disseminated and transferred horizontally via transposon-rich genomic regions [17, 53, 54]. In developing nations, it has become concerning due to the contradictory antibiotic policies [55]. Notably, WGS analysis of *K. pneumoniae* WSF99 revealed the presence of *bla*_NDM−5_ carbapenemase-encoding gene positioned within a mobile cassette, suggesting the possibility of dissemination among members of Enterobacteriaceae through HGT since the gene’s initial integration [55]. This gene was previously reported in ICU patients (82.35%) and pediatric patients (82.1%) in Egypt owing to carbapenem antibiotics consumption without adequate diagnostic sources [17, 56]. Additionally, *bla*_NDM−5_ was also predicted in the UK [57] and Lebanon [58]. Thus, the convergence of several antibiotic resistance genes in a single strain increases its pathogenicity and renders treatment more challenging.

Efflux systems have been identified in clinical bacterial isolates and have been linked to the MDR phenotypes [59]. A key feature of the MDR efflux system is its ability to extrude a broad-spectrum antimicrobial agent [60]. For instance, the RND efflux pumps belonging to *oqxA* and *oxqB* were predicted in *K. pneumoniae* WSF99 genome, which was previously reported in ST147 *K. pneumoniae* Rize-53-TR in Turkey [61]. It has been reported that the presence of OqxAB efflux pumps in clinical isolates of *K. pneumoniae* confers resistance to multiple antimicrobial agents such as chloramphenicol, tigecycline, quinolones, quinoxalines, and nitrofurantoin [62]. Moreover, previous studies reported the occurrence of *oqxA* and *oxqB* genes located on chromosomes, suggesting that the genome of *K. pneumoniae* is a possible reservoir of these two genes [63, 64]. Moreover, AcrAB efflux pump was also detected in the WSF99 genome, which was previously reported in MDR *K. pneumoniae* strains isolated from patients in Cairo, Egypt [65].

In clinical settings, hospital trash and surfaces treated with antimicrobial coatings containing metals contribute significantly to the high metal selection pressure, making clinical pathogens attractive for heavy metal resistance [66]. An earlier investigation revealed that widespread heavy metals in clinical environments have been associated with the acquisition of extrinsic resistance determinants, which contribute to the spread of ARGs [67, 68]. The occurrence of heavy metal resistance genes within the WSF99 genome enhances survival capabilities in hospital settings. Meanwhile, a previous study reported the detection of heavy metal resistance genes (*sil* and *ter*) in *K. pneumoniae* isolated from ICU patients in Egypt [17].

Investigating the virulome more in depth through WGS plays an essential role in determining the severity of infection caused by clinical strains of *K. pneumoniae* [69]. Strain WSF99 revealed the presence of *iuc*, *rmpA* and *rmpA2* which are predictive markers of hypervirulent *K. pneumoniae* [13, 70]. Unlike our results, previous studies reported the presence of wzi64 allele type in ST147 clones [71]. Otherwise, the presence of capsular polysaccharide gene clusters *fimABCDEFGHIK* and *mrkABCDFHI* in WSF99 genome is essential for the establishment of infection and bacterial evasion of the host immune response [14]. Furthermore, the presence of enterobactin synthetase gene clusters *iucABCD* and *entABCDEFS* in WSF99 genome tends to restrict iron bioavailability, a traditional host defense mechanism against bacterial invasion [71]. These iron uptake systems that capture siderophores are one of the strategies used by bacteria to boost their pathogenicity potential [72]. Type I and type III fimbriae were predicted in the WSF99 genome, which were critical in initiating the adhesion process of the bacteria to the host and biofilm formation, which agrees with other studies [73, 74].

Plasmids, IS, ICEs and prophages are genetic mobile elements that present a serious clinical threat in the dissemination of virulence and resistance genes via HGT [75, 76]. The Egyptian isolates, like many other *K. pneumoniae*, harbor plasmids that encode heavy metal and/or ARG [16]. Notably, WSF99 strain revealed the presence of IncFIB (pQil) plasmid replicon associated with IS element IS*Kpn25* that was previously observed in carbapenem- and colistin-resistant *K. pneumoniae* [77]. Moreover, IS*Kpn26* is associated with IncR plasmid, suggesting that this plasmid is receptive to IS element uptake and maintenance [78]. Insertion sequence IS*26* was the most frequent in the WSF99 genome, which participated in the mobilization of ARG and played a critical role in the evolution of MDR phenotypes [79]. Additionally, the genome analysis of the WSF99 revealed the prevalence of Klebsi_ST147_VIM1phi7.1and Pseudo_phiPSA1 phages, which play a major role in the virulence and evolution of pathogenic bacteria [80].

## Conclusions

This study provides insights into the antimicrobial resistance profile and virulence in the genome sequence of *K. pneumoniae* WSF99 clone ST147. This MDR isolate revealed the presence of different classes of β-lactamase and carbapenemase antibiotic resistant genes. The comprehensive genomic data obtained by WGS highlights the severity of health challenges caused by the emergence of MDR-KP and contributes to the worldwide efforts to tack antibiotic resistance. Moreover, the study highlights the significance of regular monitoring to prevent infections in Egypt.

## Data Availability

Sequence data that support the finding of this study have been deposited in the he National Biotechnology Information Center (NCBI) GenBank database with the primary accession code JAWIZL000000000.

## Abbreviations

AMR: Antimicrobial Resistance
AST: Antimicrobial Susceptibility Testing
ARG: Antibiotic Resistance Gene
bla: Beta Lactamase
CDS: Coding Sequence
CGE: Center for Genomic Epidemiology
CLSI: Clinical and Laboratory Standards Institute
COG: Cluster Of Orthologous Groups
ESBL: Extended Spectrum β-lactamase
GBDP: Genome BLAST Distance Phylogeny
HGT: Horizontal Gene Transfer
ICE: Integrative and Conjugative Elements
ICU: Intensive Care Unit
IS: Insertion Sequence
KEGG: Kyoto Encyclopedia of Genes and Genomes
MDR: Multidrug Resistant
MGE: Mobile Genetic Element
MIC: Minimum Inhibitory Concentration
MLST: Multi-locus Sequence Typing
PGAP: Prokaryotic Genome Annotation Pipeline
RND: Resistance Nodulation Division
ST: Sequence Type
TYGS: Type (Strain) Genome Server
WGS: Whole Genome Sequencing
WHO: World Health Organization
